# Synthesis of Peptoids Containing Multiple *N*htrp and *N*trp Residues: A Comparative Study of Resin, Cleavage Conditions and Submonomer Protection

**DOI:** 10.3389/fchem.2020.00370

**Published:** 2020-04-29

**Authors:** Abdullah Lone, Anis Arnous, Paul Robert Hansen, Biljana Mojsoska, Håvard Jenssen

**Affiliations:** ^1^Department of Science and Environment, Roskilde University, Roskilde, Denmark; ^2^Department of Drug Design and Pharmacology, Faculty of Health and Medical Sciences, University of Copenhagen, Copenhagen, Denmark

**Keywords:** peptoids, tryptamine, submonomer synthesis, solid phase synthesis, tryptophan

## Abstract

Peptoids hold status as peptide-mimetics with versatile biological applications due to their proteolytic stability and structural diversity. Among those that have been studied in different biological systems, are peptoids with dominant balanced hydrophobic and charge distribution along the backbone. Tryptophan is an important amino acid found in many biologically active peptides. Tryptophan-like side chains in peptoids allow H-bonding, which is absent from the parent backbone, due to the unique indole ring. Furthermore, the rigid hydrophobic core and flat aromatic system influence the positioning in the hydrocarbon core and allows accommodating tryptophan-like side chains into the interfacial regions of bacterial membranes and causing bacterial membrane damage. Incorporating multiple tryptophan-like side chains in peptoids can be tricky and there is a lack of suitable, synthetic routes established. In this paper, we investigate the synthesis of peptoids rich in *N*htrp and *N*trp residues using different resins, cleavage conditions, and unprotected as well as *tert*-butyloxycarbonyl-protected amines suitable for automated solid-phase submonomer peptoid synthesis protocols.

## Introduction

Modern medicine has blossomed as a result of the introduction of antimicrobial drugs in the early 1940s, however, over-prescription and misuse of these lifesaving drugs have tumbled modern societies into a post-antibiotic era. Novel antimicrobial drugs are in high demand and much attention has been given to naturally occurring antimicrobial peptides (AMPs) and derivatives thereof. AMPs are characterized as short (12–50 amino acids), cationic (net charge +2 to +9), and amphiphilic (Jenssen et al., 2006). They are multifunctional biomolecules, e.g., possessing antibacterial (Bechinger and Gorr, 2017), antiviral (Jenssen, 2005), antifungal (Lacerda et al., 2014) properties, stimulating host immune cell responses (Nijnik et al., 2010; Steinstraesser et al., 2012), and affecting cell migration (Andrea et al., 2018; Mouritzen et al., 2018; Vang Mouritzen and Jenssen, 2018). The clinical success of AMPs may have been limited by disadvantages, such as susceptibility to proteolytic degradation, pH and/or salinity-dependent activity. This has fueled the development of peptidomimetics such as peptoids (*N*-substituted glycines) (Zuckermann et al., 1992; Chongsiriwatana et al., 2008; Godballe et al., 2011; Molchanova et al., 2017), a class of molecules mimicking the features of AMPs while being designed as proteolytically stable entities.

Both AMPs and antimicrobial peptidomimetics have been widely studied for their antibacterial properties in solution tests, initially tested for activity toward planktonic bacteria but lately also toward microbial biofilms (Kapoor et al., 2011; Secker et al., 2015; Batoni et al., 2016; De La Fuente-Núñez et al., 2016; Chung and Khanum, 2017; Wang et al., 2017; Andrea et al., 2018; Lee et al., 2018). Peptoid macrocycles have also been studied as antimicrobial agents that show remarkable selectivity toward microbial over eukaryotic cells (Shin et al., 2007; Huang et al., 2012). A generalized conclusion that can be drawn from three decades of structure-activity studies of AMPs is that active AMPs predominantly need a balance of cationic residues (arginine and/or lysine) and about 50% hydrophobic residues. In addition, increased hydrophobicity is often shown to be correlated with increased mammalian cell toxicity (Mojsoska et al., 2015; Turkett and Bicker, 2017). Amongst the hydrophobic residues particularly tryptophan appears to be crucial and often position dependent for the activity. The literature is full of examples where tryptophan is more or less conserved in certain positions in some AMP families (Andreu et al., 1985) e.g., in N-terminal position 1 or 2 in many cecropins or appears in patterns with the cationic residues i.e., Lys-Trp or Arg-Trp (Bang et al., 2010). Systematic substitution analysis studies have been carried out for numerous AMPs and generally, it is concluded through alanine scans that tryptophan is important or crucial for antimicrobial activity (Strøm et al., 2000). The hydrophobic bulkiness of the tryptophan side-chain has also importance for the activity and can only be replaced by certain hydrophobic non-natural building blocks (Ryge et al., 2004). One likely explanation is that the aromatic system in the tryptophan side-chain generates a cloud of negative charge which can interact with the positively charged guanidinium group of arginine (Aliste et al., 2003). It has been suggested that upon interaction with the bacterial membrane the guanidinium group favors interaction with negatively charged lipopolysaccharide or lipoteichoic acid, thus exposing the negatively charged flanks of tryptophan which inserts into the lipid interface layer, an interaction which is stabilized by the positively charged choline head groups (Aliste et al., 2003). Additionally, the bulky nature of tryptophan and hydrogen-bonding potential is important for its membrane-disruptive activities (Vogel et al., 2002), though the hydrogen bonding of tryptophan diminishes upon insertion into the core of the membrane bilayer (Yau et al., 1998). It is also apparent that positioning of the tryptophan mimics in antimicrobial peptoids are of importance for the biological activity (Mojsoska et al., 2015) and also influencing on the antibacterial mode of action (Mojsoska et al., 2017; Saporito et al., 2019). Thus, the development of novel peptidomimetic drug candidates and in particular peptoids would benefit from building blocks mimicking the physicochemical features of the tryptophan side chain. Special efforts to incorporate heterocycle-containing side chains into peptoid have been made, including by protection of the heterocycles' nucleophilic sites (Uno et al., 1999; Burkoth et al., 2003). However, tryptophan-mimicking side chains are still challenging to incorporate into peptoids, even applying these modifications. Considering its importance in AMP research, there is a need for strategies, which would enable easy, low cost, and predictable incorporation of such building blocks into future peptoid libraries using automated technologies.

In the present work, we investigate the synthesis of peptoids containing multiple *N*htrp and *N*trp residues, using different (1) resins (2) cleavage conditions, and (3) protected and unprotected sub-monomer amines.

## Materials and Methods

### Chemicals and Instruments

TentaGel S RAM (0.22 mmol/g) were purchased from Rapp Polymere GmbH. Rink Amide MBHA resin (0.65 mmol/g) were purchased from Novabiochem^1^ Tryptamine, N-Boc-1,4-butanediamine, and benzylamine were purchased from Sigma-Aldrich. 1-Boc-Tryptamine (1H-Indol-3yl) methanamine, Boc-(1H-Indol-3yl) methanamine were purchased from Combi-Blocks. Trifluoroacetic acid (TFA) and N-methyl-2-pyrrolidone (NMP) were purchased from VWR. Piperidine and bromoacetic acid were purchased from Merck. N,N-diisopropylcarbodiimide (DIC), triisopropylsilane (TIPS) and anisole were purchased from Sigma-Aldrich. Dimethylformamide (DMF), dichloromethane (DCM), acetonitrile were from Th.Geyer. All reagents and solvents were used without further purification. Disposable reactors (5 mL polypropylene) fitted with a PTFE filter were acquired from Fa. Gerhardt, Kassel Germany.

### General Procedure for Peptoid Synthesis

#### Manual Peptoid Synthesis

**Figure 1:**
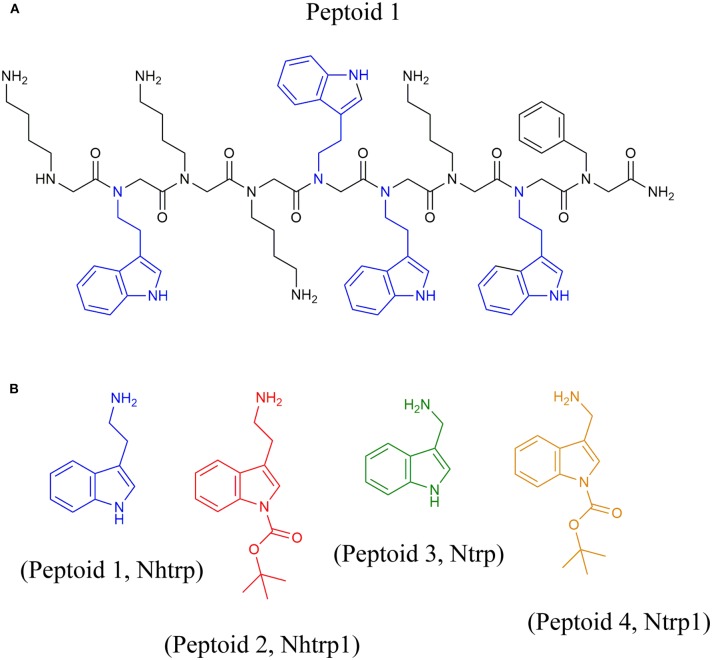
**(A)** Chemical structure of peptoid 1 (GN-2 Npm_9_ peptoid) (Mojsoska et al., 2015). **(B)** Chemical structures of 4 indole containing amines used in the design and synthesis of peptoids 1–4 at positions 2, 4, 5, and 7. Tryptamine (blue), 1-Boc-Tryptamine (red), (1H-Indol-3yl) methanamine (green), and Boc-(1H-Indol-3yl) methanamine (orange). The monomeric units corresponding to the four different amines are abbreviated as *N*htrp, *N*htrp1, *N*trp, and *N*trp1.

**Scheme 1:**
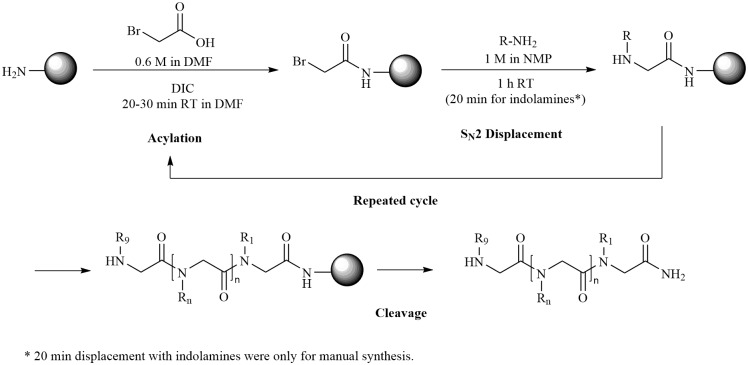
Solid-phase sub monomer peptoid synthesis of indole rich peptoids. * 20 min displacement with indolamines was used only for manual synthesis protocol.

Peptoid 1 (GN2-Npm_9_, Figure 1A) was synthesized using standard submonomer solid-phase synthesis (see Scheme 1) as described by Tran et al. (2011) with minor modifications. The peptoid was synthesized manually (100 mg scale) in disposable 5 mL polypropylene reactors fitted with a PTFE filter using either a MBHA resin (0.65 mmol/g) or TentaGel S RAM resin (0.22 mmol/g). The resin was swelled in DMF for 2 h at room temperature, followed by Fmoc deprotection by 20% piperidine in DMF for 2 × 10 min. The resin was washed with DMF (4 × 2 min with 2–3 mL). The acylation step was performed using 0.6 M bromoacetic acid in DMF and 86 μL DIC in DMF for 20 min (30 min for first acylation step). Displacement was achieved through the addition of 1 M amine of interest in NMP for 1 h (20 min for tryptamine) at room temperature.

#### Solid-Phase Automated Peptoid Synthesis

The setup from the manual peptoid synthesis was transferred to a solid-phase automated peptide synthesizer (ResPep SL; Intavis Bioanalytical Instruments AG) for synthesis of peptoids 1–4 in microcolumns (20 mg resin in each microcolumn) using either a MBHA resin (0.65 mmol/g) or a TentaGel S RAM resin (0.22 mmol/g).

### General Procedure for Cleavage and Characterization

Peptoids were cleaved using two different cleavage conditions, A (TFA:TIPS:H_2_O, 95:2.5:2.5) and B (TFA:DCM:anisole, 49:49:2) for 30 min, shaking at room temperature. After cleavage, the cleavage cocktail solution was filtered and evaporated under N_2_ gas. The residual was dissolved in 5% acetonitrile in milli-Q water. All peptoids were analyzed using C18 Kinetex 100 × 2.1 mm 100 Å column, 40°C, linear gradient of 5–65% water (0.1% formic acid) in acetonitrile (0.1% formic acid), flow rate 0.5 mL/min on Dionex Ultimate 3000 UHPLC connected to Thermo Finnigan LTQ-XL linear ion-trap mass spectrometer. Mass detection within 250–2,000 *m/z* range was done using positive mode on a diode array detector interfaced with a HESII electro spray ion source. For percentile crude purity calculation, mass chromatograms were integrated using Peak detection ICIS algorithm using FreeStyle™ 1.5 software (Thermo Scientific). To control for impurities that come from the resin itself, samples from both MBHA and TentaGel S RAM resins cleaved with the two different cleavage conditions were analyzed in the same way as all the length mers. The data is shown in Table S3.

## Results and Discussion

Several studies have highlighted the biological relevance of the unique indole ring structure in tryptophan and tryptamine like residues. We have previously demonstrated that the tryptamine rich peptoid GN-2 Npm_9_ (Figure 1A, peptoid 1) selectively kills Gram-negative bacteria (Mojsoska et al., 2015, 2017). Synthesis of peptoids rich in indole like monomers appears challenging especially when using unprotected indole rich amine monomers. In an attempt to investigate several parameters in the solid-phase synthesis routes of indole rich peptoids, we selected peptoid 1 as a model compound. We systematically chose to alternate three parameters for synthesis to gain insight about the crude purity, side-product formed after each displacement step and use this information to identify the need of using protected indole tryptamine-like amines. These parameters were, (1) choice of resin, (2) cleavage conditions, and (3) use of four different protected and unprotected tryptamine-like amines (Figure 1B).

### Total Crude Purity of Indole Rich Peptoids Varies With Resin Choice

**Figure 2:**
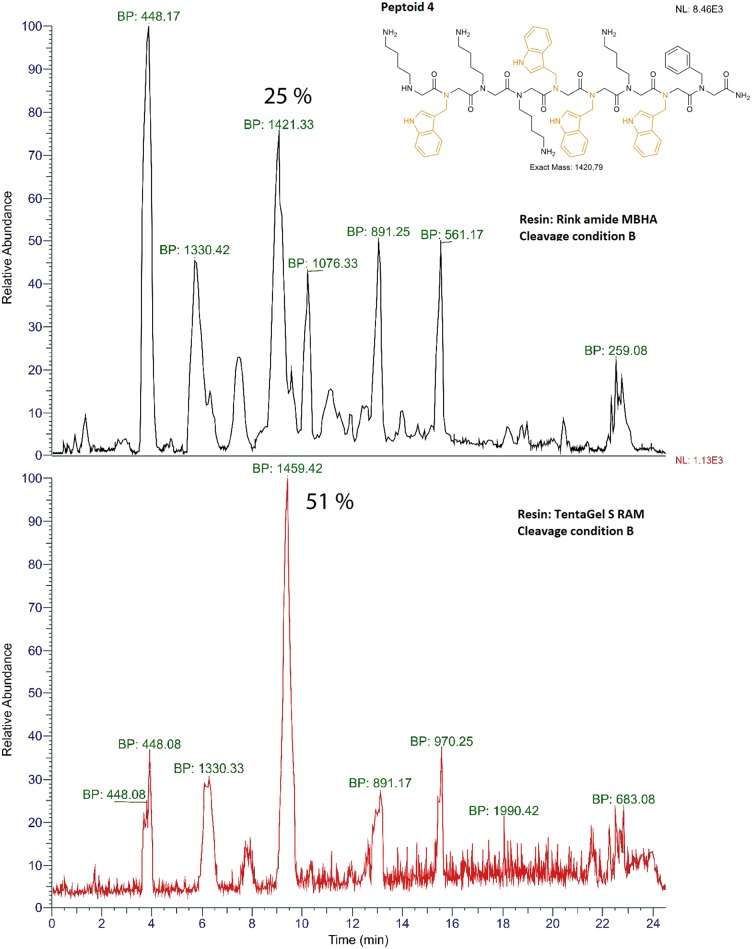
Mass chromatograms of peptoid 4 (shown) synthesized using automated solid-phase synthesizer and different resins, Rink Amide MBHA (top) and TentaGel S RAM (bottom). The most intensive *m/z* values are shown as Base Peak (BP) signals for each mass chromatogram. The chromatograms were obtained on RP-HPLC-ESI-MS using acetonitrile gradient over 24 min. Percentile area (% crude purity) for each peak was determined using Peak Detection algorithm (ICIS, FreeStyle 1.5, Thermo Scientific) where peaks with areas more than 5% were integrated. Y-axis shows local data normalization. The correct product mass was identified using observed *m/z* values corresponding to M^+^, [M+2H+]^2+^ and/or M^+^ Adduct ions. Crude product after deprotection and cleavage using condition B (TFA:DCM:anisole (v/v%, 49:49:2) from resin is shown.

Initially, we synthesized peptoid 1 using a manual solid-phase submonomer synthesis protocol employing two different C-terminal amide resins, MBHA and TentaGel S RAM. A small amount of resin was taken out for cleavage to estimate crude purity after each step of bromoacetylation and displacement. Results from crude purities (%) are shown in Table 1. The parent mass was identified using *m/z* base peak signals with the highest intensity. The overall purity of the final product across the length mers was higher for the peptoid synthesized using TentaGel S RAM resin. To exemplify, the overall purities for 2 mer were calculated to be 37 and 56%, the 4 mer sequence the 43 and 69% and for the 9 mer 38 and 78% for MBHA and TentaGel S RAM, respectively (Table 1). We then transferred the manual synthesis steps to an automated solid-phase peptide synthesizer and synthesized peptoid 1 again. In the automated synthesis set-up, TentaGel S RAM remained the resin of choice giving higher total crude purity than MBHA resin (**Figure 4** left and right, Peptoid 1). TentaGel S RAM also remains the choice of resin for peptoid 2 and 4 regardless of cleavage conditions (Table 2, Figure 2). When compared to the manual synthesis of peptoid 1 using TentaGel S RAM, the automation resulted in lower crude purities of 78 and 33%, respectively (Tables 1, 2). It's worth noting that one of the differences between the manual and automated synthesis set up was the shaking condition between each synthesis step, which is absent in the latter. Proper shaking conditions allow a higher degree of incorporation of building blocks along the peptoid chain and with this in mind longer displacement time was employed for all amines using the automated synthesis protocols (20 vs. 60 min, respectively). In the present study, TentaGel Resin performs better than Rink MBHA due to lower loading (0.22 and 0.65 mmol/g, respectively) and better swelling properties in DMF. The Rink MBHA resin is a polystyrene a polymer cross-linked with 1% of divinylbenzene, while the TentaGel S RAM resin has polyethyleneglycol grafted (50–70%) to low cross-linked polystyrene. Furthermore, TentaGel S RAM swells better than Rink MBHA in DMF, 5 vs. 4 mL/g, respectively (Shelton and Jensen, 2013).

**Table 1:** Crude purity and mass spectrometric analyses from a manual synthesis of peptoid 1 (Figure 1A) using two different resin (rink amide MBHA and TentaGel S RAM resins) and cleavage condition A.

**Total crude purity (%)**^****a****^			**Corresponding** ***m/z*** **base peak signal**^****b****^	
**Peptoid length mer**	**MBHA^**c**^**	**TentaGel S RAM^**d**^**	**MBHA**	**TentaGel S RAM**
2 mer	37	56	387.25 (365.25)	387.25 (365.17)
3 mer	27	43	515.4 (493.33)	515.4 (493.42)
4 mer	43	69	693.5 (715.58)	693.5
5 mer	66	88	893.67	915.67 (893.58, 931.67)
6 mer	68	94	1021.75 (1043.83)	1021.67
7 mer	56	85	1149.83 (1171.83)	1149.75 (1171.92)
8 mer	45	100	1349.9 (1371.92, 1387.92)	1371.92 (1349.75, 1387.92)
9 mer	38	78	1500.08 (1516.08)	1500.08

a *Analytical RP-HPLC-ESI-MS conditions: C18 Kinetex 100 × 2.1 mm 100 Å column, 40°C, linear gradient of 5–65% water in acetonitrile (0.1% HCOOH), flow rate 0.5 mL/min. Crude purity calculated with peak detection integration method ICIS (FreeStyle 1.5, Thermo Scientific), where peaks with areas more than 5% were integrated. Percentile crude purity of each length mer is shown independently of the previous length mer purity*.

b *Observed masses of [M+2H+]^2+^, M+ H^+^ or Na^+^, K^+^ adduct*.

c *Rink amide MBHA (loading: 0.65 mmol/g)*.

d *TentaGel S RAM (loading: 0.22 mmol/g)*.

**Table 2:** Total crude purity (%) and mass spectrometric analyses for all peptoids 1–4 synthesized on automated solid-phase peptide synthesizer using two different resins and two cleavage conditions A and B.

**Total crude purity (%)**^****a****^					**Corresponding** ***m/z*** **base peak signals**^****b****^			
	**MBHA**		**TentaGel S RAM**		**MBHA**		**TentaGel S RAM**	
**Peptoid**	**A^**c**^**	**B^**d**^**	**A^**c**^**	**B^**d**^**	**A^**c**^**	**B^**d**^**	**A^**c**^**	**B^**d**^**
1	6	6	33	53	1515.50 (1499.58, 1478.33)	1515.50 (1499.33)	1499.5	1515.42 (1500.50, 1499.5)
2	20	26	34	61	1515.50 (1499.58, 1477.08, 739.33)	1515.50 (1499.50, 1477.42, 739.83, 758.83)	1499.58 (1515.50)	1516.5
3^e^	/	/	/	/	/	/	/	/
4	6	25	26	51	1443.83 (1421.25)	1421.33 (1443.42, 1459.42)	1460.42 (1443.50)	1459.42 (1443.42)

a *Analytical RP-HPLC-ESI-MS conditions: C18 Kinetex 100 × 2.1 mm 100 Å column, 40°C, linear gradient of 5-65% water in acetonitrile (0.1% HCOOH), flow rate 0.5 mL/min. Crude purity calculated with peak detection integration method ICIS (FreeStyle 1.5, Thermo Scientific) where peaks with areas more than 5% were integrated*.

b *Observed masses of [M+2H+]^2+^, M+ H^+^ or Na^+^, K^+^ adduct*.

c *[TFA:TIPS:H_2_O (v/v%, 95:2.5:2.5)], 30 min, RT*.

d *[TFA:DCM:anisole (v/v%, 49:49:2)], 30 min, RT*.

e *We did not obtain any of the desired peptoid product*.

### Cleavage Conditions Influence the Final Crude Purity of Peptoids

**Figure 3:**
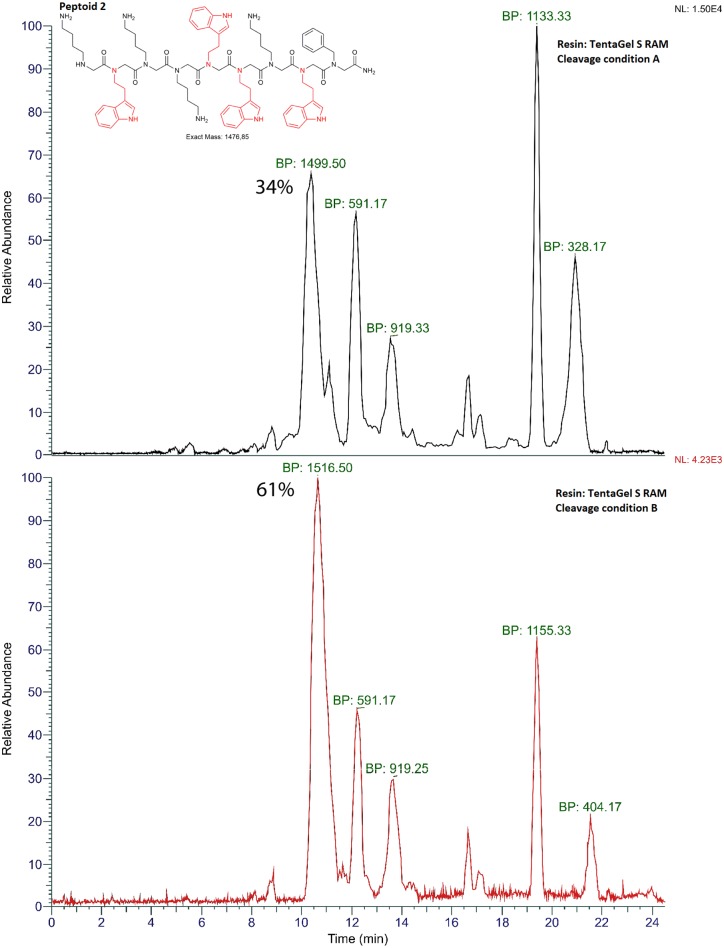
Mass chromatograms of peptoid 2 (shown) synthesized using automated solid-phase synthesizer, TentaGel S RAM resin, cleaved with two different cleavage conditions, A TFA:TIPS:H_2_O (95:2.5:2.5) and B TFA:DCM:anisole (49:49:2). The most intensive *m/z* values are shown as Base Peak (BP) signals for each mass chromatogram. The chromatograms were obtained on RP-HPLC-ESI-MS using acetonitrile/water gradient over 24 min. Percentile area (% crude purity) for each peak was determined using Peak Detection algorithm (ICIS, FreeStyle 1.5, Thermo Scientific) where peaks with areas more than 5% were integrated. Y-axis shows local data normalization. The product was identified using observed *m/z* values corresponding to M^+^, [M+2H+]^2+^ and/or M^+^ adduct ions. Crude after deprotection and cleavage using cleavage condition A TFA:TIPS:H_2_O (v/v%, 95:2.5:2.5) or B TFA:DCM:anisole (v/v%, 49:49:2) from resin is shown.

**Scheme 2:**
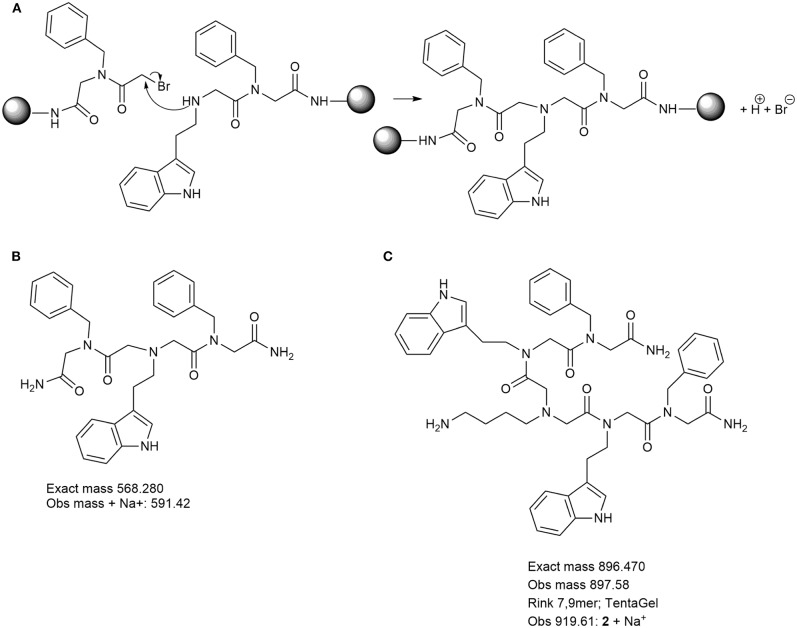
**(A)** Mechanism of formation of terminated cross-linked peptoids. **(B,C)** Chemical structures and masses (calculated and observed) for some of the major side product impurities from synthesis of Peptoids 1 length mers in Table S1. Product B possibly generated via S_N_2 reaction of bromoacetylated *N*pm C-terminal residue and the backbone amine of *N*htrp-*N*pm. This product was identified in all length mers analysis for Peptoid 1 synthesized using MBHA rink amide resin and in 2–4, 7, and 9 mer analysis for the same synthesized using TentaGel S RAM resin. Product C was the second major side-product with a mass of 897.58 *m/z* that results from the cross-linking of a bromoacetylated *N*htrp-*N*pm with *N*lys-*N*htrp-*N*pm. This product was only identified in 4–6 and 8 mer analysis for Peptoid 1 synthesized using MBHA rink amide resin.

**Scheme 3:**
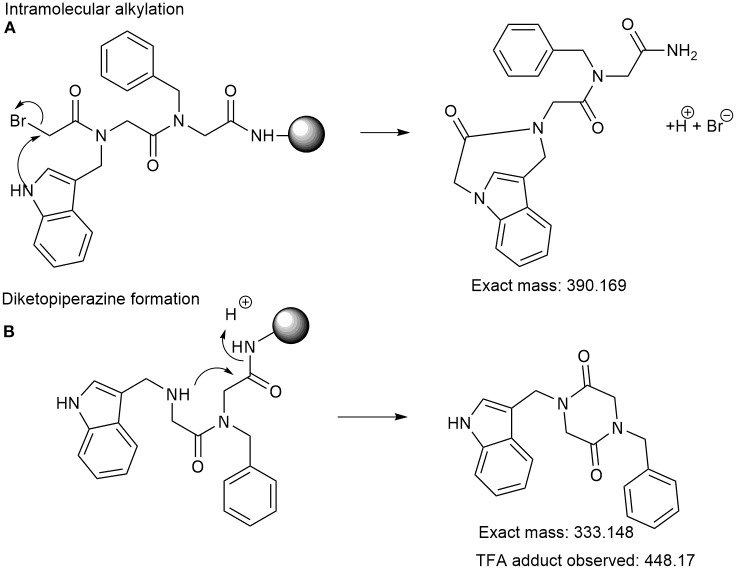
Two possible termination mechanisms for peptoid 3 incorporating unprotected tryptophan-like amine residue *N*trp. **(A)** Intramolecular alkylation. **(B)** Diketopiperazine formation. Exact mass and the mass of the observed (TFA adduct) compounds are shown.

To further investigate the low crude purity observed when using MBHA resin but also TentaGel S RAM resin when the peptoid was synthesized using an automated set up, we inspected the effects of using different cleavage cocktails as an additional parameter. In the literature several protocols suggest cleaving conditions depending on the side chain functionality and the choice of resin when using solid-phase synthesis protocols (Bernatowicz et al., 1989; Sherrington, 1990; Walker, 2012; Novabiochem). Peptoids were cleaved under two different cleavage conditions, A (TFA:TIPS:H_2_O; 95:2.5:2.5) and B (TFA:DCM:anisole, 49:49:2) for 30 min. To minimize undesired acylation upon generation of cleaved peptoid products, especially where indole side chain in tryptamine and analogs amines can react with electrophiles under acidic conditions, we used anisole and triisopropylsilane (TIPS) as carbocation scavengers (Pearson et al., 1989). Data from total crude purity for each of the cleavage condition are shown in Table 2. Overall, the data suggest that using less acidic cleavage conditions (condition B) improved the final crude purity (Table 2, Figure 3). In addition to this, for each step in the manual synthesis and at the final product (9 mer) of the automated synthesis, when present, several traces (impurities) were analyzed (Table S2) and possible structures were suggested (Scheme 2, 3). By doing structural analysis of the impurities, we could identify several products and investigate whether those were generated during the synthesis or they occurred during cleavage of the peptoids from the resin. In the manual synthesis of peptoid 1 we observed two major side-products (Scheme 2) both resulting from cross-linking. The first side product with an observed mass of 591.42 *m/z* was present in all Rink syntheses and TentaGel 2, 3, 4, 7, and 9 (Scheme 2B, Table S1). A bromoacetylated *N*pm C-terminal residue reacts with the backbone amine of *N*htrp-*N*pm in a S_N_2 reaction (Scheme 2A). The second major side-product with a mass of 897.58 *m/z* results from the cross-linking of a bromoacetylated *N*htrp-*N*pm with *N*lys-*N*htrp-*N*pm. This *m/z* was present in Rink syntheses 4, 5, 6, and 8 but not at all in syntheses using TentaGel (Scheme 2C, Table S1). This may be explained by the higher loading of the Rink resin MBHA resin (0.65 mmol/g) vs. TentaGel S RAM resin (0.22 mmol/g).

### Protecting the Indole Ring in Tryptamine and (1H-Indol-3yl) Methanamine Improved the Final Crude Purity

**Figure 4:**
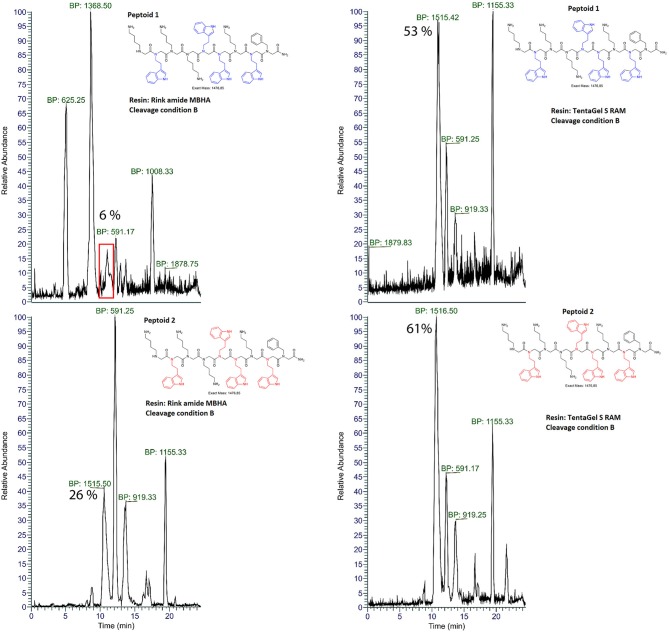
Mass chromatograms of peptoid 1 with *N*htrp (top) and peptoid 2 with *N*htrp1 building blocks (bottom) synthesized using automated solid-phase synthesizer, Rink Amide MBHA (left) and TentaGel S RAM resins (right). The most intensive *m/z* values are shown as Base Peak (BP) signals for each mass chromatogram. The chromatograms were obtained on RP-HPLC-ESI-MS using acetonitrile gradient over 24 min. Percentile area (% crude purity) for each peak was determined using Peak Detection algorithm (ICIS, FreeStyle, Thermo Scientific) where peaks with areas more than 5% were integrated. Y-axis shows local data normalization. The correct product mass was identified using observed *m/z* values corresponding to M^+^, [M+2H+]^2+^ and/or M^+^ adduct ions. Crude after deprotection and cleavage using cleavage condition B (TFA:DCM:anisole (v/v%, 49:49:2) from resin is shown.

Rink amide linkers and Boc protecting groups require presence of TFA in a standardized cleavage cocktail. In presence of high TFA concentrations, formation of highly reactive cationic species from the protecting groups and the resin-linkers are generated that can react and consequently modify electron-rich side chain such is the indole ring in tryptophan, tryptamine, and analogs. To explore the contribution of possible side chain reaction of the indole ring in tryptamine and its analogs to the number of observed impurities, we looked at use of both Boc-protected and unprotected indole containing amines in peptoid synthesis as our third parameter (Figure 1B). Systematically incorporating these amines along the peptoid chain at positions 2, 4, 5, and 8 we generated 4 peptoids. We hypothesized that the low observed purity could be associated with side reaction arising from the unprotected indole ring during the synthesis but also during cleaving of the product. We investigated whether the protection of the indole e.g., retards decomposition as scavengers can sometimes also reduce the indole (Pearson et al., 1989). Our data showed that in some cases the total crude purity is significantly higher when using Boc protected amine (Figure 4).

In the synthesis of peptoid 3, using the unprotected submonomer *N*trp ((1*H*-indol-3-yl)methaneamine) we did not obtain any of the desired peptoid product. Previously, Zuckermann and coworkers have reported that a few submonomers may undergo two side-reactions in peptoid synthesis (Figliozzi et al., 1996). The main chain bromoacetic acid group may react with an amino group three or four atoms away from the backbone amide (Scheme 3A). The submonomer (2-aminomethyl) benzimidazole falls into this category and is closely related to the *N*trp submonomer. The second reaction is the formation of *N*-substituted diketopiperazines (Scheme 3B). We observed the *N*-substituted diketopiperazine TFA adduct with an *m/z* of 448.17, although this was not the main side-product (Scheme 3B).

## Conclusion

The aim of this work was to gain insight into the synthesis of peptoids rich in tryptamine (*N*trp) and ((1*H*-indol-3-yl)methaneamine) (*N*htrp) residues, using different protocols. Using peptoid 1 (GN-2 Npm_9_) as a model compound, we investigated three parameters (1) resins (MBHA or TentaGel S RAM; (2) cleavage conditions with TFA:TIPS:H_2_O (95:2.5:2.5) or TFA:DCM:anisole (49:49:2); and (3) use of unprotected as well as Boc-protected tryptamine and Boc-protected ((1*H*-indol-3-yl)methaneamine). We found that TentaGel Resin (loading 0.22 mmol/g) resulted in fewer side-products compared to the MBHA resin (loading 0.65 mmol/g). Furthermore, we found that TFA:DCM:anisole (49:49:2) for 30 min is sufficient for cleavage and gives better results than TFA:TIPS:H_2_O (95:2.5:2.5). Finally, synthesis of peptoid 1 was successful using both Boc-protected and unprotected submonomers, showing that protection of tryptamine is not required. However, Boc-protection of ((1*H*-indol-3-yl) methaneamine) is crucial, since we observed no desired product when using the unprotected submonomer. The results from this study show the contribution of impurities that can be found at each displacement step in the submonomer peptoid synthesis protocol. The results from this study will help researchers synthesize peptoids containing multiple *N*htrp and *N*trp residues for various applications such as discovery of new antimicrobial agents, protein antagonists (Quintanar-Audelo et al., 2007) or labeling with novel indolamines (Antos et al., 2009).

## Data Availability Statement

All datasets generated for this study are included in the article/Supplementary Material.

## Author Contributions

AL, PH, BM, and HJ: conceptualization, writing-original draft preparation, writing-review, and editing. AL, AA, and BM: investigation. PH, BM, and HJ: supervision.

## Conflict of Interest

The authors declare that the research was conducted in the absence of any commercial or financial relationships that could be construed as a potential conflict of interest.

## Footnotes

^1^Novabiochem Fmoc resin cleavage protocols. (2019). Available online at: https://www.emdmillipore.com/Web-US-Site/en_CA/-/USD/ShowDocument-Pronet?id=201503.030.

## References

[B1] Aliste M. P. MacCallum J. L. Tieleman D. P. (2003). Molecular dynamics simulations of pentapeptides at interfaces: salt bridge and cation–π interactions. Biochemistry 42, 8976–8987. 10.1021/bi027001j 12885230

[B2] Andrea A. Molchanova N. Jenssen H. (2018). Antibiofilm peptides and peptidomimetics with focus on surface immobilization. Biomolecules 8:27. 10.3390/biom8020027 29772735 PMC6022873

[B3] Andreu D. Steiner H. Boman H. G. (1985). N-Terminal analogues of cecropin a: synthesis, antibacterial activity, and conformational properties. Biochemistry 24, 1683–1688. 10.1021/bi00328a017 3924096

[B4] Antos J. M. McFarland J. M. Iavarone A. T. Francis M. B. (2009). Chemoselective tryptophan labeling with rhodium carbenoids at mild pH. J. Am. Chem. Soc. 131, 6301–6308. 10.1021/ja900094h 19366262 PMC2722835

[B5] Bang J. K. Nan Y. H. Lee E. K. Shin S. Y. (2010). A novel Trp-rich model antimicrobial peptoid with increased protease stability. Bull. Korean Chem. Soc. 31, 2509–2513. 10.5012/bkcs.2010.31.9.2509

[B6] Batoni G. Maisetta G. Esin S. (2016). Antimicrobial peptides and their interaction with biofilms of medically relevant bacteria. Biochim. Biophys. Acta Biomembr. 1858, 1044–1060. 10.1016/j.bbamem.2015.10.013 26525663

[B7] Bechinger B. Gorr S. U. (2017). Antimicrobial peptides: mechanisms of action and resistance. J. Dent. Res. 96, 254–260. 10.1177/0022034516679973 27872334 PMC5298395

[B8] Bernatowicz M. S. Daniels S. B. Köster H. (1989). A comparison of acid labile linkage agents for the synthesis of peptide C-terminal amides. Tetrahedron Lett. 30, 4645–4648. 10.1016/S0040-4039(01)80764-4

[B9] Burkoth T. S. Fafarman A. T. Charych D. H. Connolly M. D. Zuckermann R. N. (2003). Incorporation of unprotected heterocyclic side chains into peptoid oligomers via solid-phase submonomer synthesis. J. Am. Chem. Soc. 125, 8841–8845. 10.1021/ja0352101 12862480

[B10] Chongsiriwatana N. P. Patch J. A. Czyzewski A. M. Dohm M. T. Ivankin A. Gidalevitz D.. (2008). Peptoids that mimic the structure, function, and mechanism of helical antimicrobial peptides. Proc. Natl. Acad. Sci. U.S.A. 105, 2794–2799. 10.1073/pnas.0708254105 18287037 PMC2268539

[B11] Chung P. Y. Khanum R. (2017). Antimicrobial peptides as potential anti-biofilm agents against multidrug-resistant bacteria. J. Microbiol. Immunol. Infect. 50, 405–410. 10.1016/j.jmii.2016.12.005 28690026

[B12] De La Fuente-Núñez C. Cardoso M. H. De Souza Cândido E. Franco O. L. Hancock R. E. W. (2016). Synthetic antibiofilm peptides. Biochim. Biophys. Acta 1858, 1061–1069. 10.1016/j.bbamem.2015.12.015 26724202 PMC4809770

[B13] Figliozzi G. M. Goldsmith R. Ng S. C. Banville S. C. Zuckermann R. N. (1996). Synthesis of N-substituted glycine peptoid libraries. Methods Enzymol. 267, 437–447. 10.1016/S0076-6879(96)67027-X 8743331

[B14] Godballe T. Nilsson L. L. Petersen P. D. Jenssen H. (2011). Antimicrobial β-peptides and α-peptoids. Chem. Biol. Drug Des. 77, 107–116. 10.1111/j.1747-0285.2010.01067.x 21266014

[B15] Huang M. L. Shin S. B. Y. Benson M. A. Torres V. J. Kirshenbaum K. (2012). A comparison of linear and cyclic peptoid oligomers as potent antimicrobial agents. ChemMedChem. 7, 114–22. 10.1002/cmdc.201100358 21990117

[B16] Jenssen H. (2005). Anti herpes simplex virus activity of lactoferrin/lactoferricin - an example of antiviral activity of antimicrobial protein/peptide. Cell. Mol. Life Sci. 62, 3002–3013. 10.1007/s00018-005-5228-7 16261265 PMC11139097

[B17] Jenssen H. Hamill P. Hancock R. E. W. (2006). Peptide antimicrobial agents. Clin. Microbiol. Rev. 19, 491–511. 10.1128/CMR.00056-05 16847082 PMC1539102

[B18] Kapoor R. Wadman M. W. Dohm M. T. Czyzewski A. M. Spormann A. M. Barron A. E. (2011). Antimicrobial peptoids are effective against Pseudomonas aeruginosa biofilms. Antimicrob. Agents Chemother. 55, 3054–3057. 10.1128/AAC.01516-10 21422218 PMC3101385

[B19] Lacerda A. F. Vasconcelos É. A. R. Pelegrini P. B. Grossi de Sa M. F. (2014). Antifungal defensins and their role in plant defense. Front. Microbiol. 5:116. 10.3389/fmicb.2014.00116 24765086 PMC3980092

[B20] Lee J. Kang D. Choi J. Huang W. Wadman M. Barron A. E.. (2018). Effect of side chain hydrophobicity and cationic charge on antimicrobial activity and cytotoxicity of helical peptoids. Bioorganic Med. Chem. Lett. 28, 170–173. 10.1016/j.bmcl.2017.11.034 29198866

[B21] Mojsoska B. Carretero G. Larsen S. Mateiu R. V. Jenssen H. (2017). Peptoids successfully inhibit the growth of gram negative *E. coli* causing substantial membrane damage. Sci. Rep. 7:42332. 10.1038/srep42332 28195195 PMC5307948

[B22] Mojsoska B. Zuckermann R. N. Jenssen H. (2015). Structure-activity relationship study of novel peptoids that mimic the structure of antimicrobial peptides. Antimicrob. Agents Chemother. 59, 4112–4120. 10.1128/AAC.00237-15 25941221 PMC4468694

[B23] Molchanova N. Hansen P. R. Franzyk H. (2017). Advances in development of antimicrobial peptidomimetics as potential drugs. Molecules. 22:1430. 10.3390/molecules22091430 28850098 PMC6151827

[B24] Mouritzen M. V. Abourayale S. Ejaz R. Ardon C. B. Carvalho E. Dalgaard L. T.. (2018). Neurotensin, substance P, and insulin enhance cell migration. J. Pept. Sci. 24, 1–10. 10.1002/psc.3093 29938867

[B25] Nijnik A. Madera L. Ma S. Waldbrook M. Elliott M. R. Easton D. M.. (2010). Synthetic cationic peptide IDR-1002 provides protection against bacterial infections through chemokine induction and enhanced leukocyte recruitment. J. Immunol. 184, 2539–2550. 10.4049/jimmunol.0901813 20107187

[B26] Pearson D. A. Blanchette M. Lou Baker M. Guindon C. A. (1989). Trialkylsilanes as scavengers for the trifluoroacetic acid deblocking of protecting groups in peptide synthesis. Tetrahedron Lett. 30, 2739–2742. 10.1016/S0040-4039(00)99113-5

[B27] Quintanar-Audelo M. Fernández-Carvajal A. Van Den Nest W. Carreño C. Ferrer-Montiel A. Albericio F. (2007). Design and synthesis of indole-based peptoids as potent noncompetitive antagonists of transient receptor potential vanilloid 1. J. Med. Chem. 50, 6133–6143. 10.1021/jm070612v 17985859

[B28] Ryge T. S. Doisy X. Ifrah D. Olsen J. E. Hansen P. R. (2004). New indolicidin analogues with potent antibacterial activity. J. Pept. Res. 64, 171–185. 10.1111/j.1399-3011.2004.00177.x 15485555

[B29] Saporito P. Mojsoska B. Løbner Olesen A. Jenssen H. (2019). Antibacterial mechanisms of GN-2 derived peptides and peptoids against *Escherichia coli*. Biopolymers 110:e23275. 10.1002/bip.23275 30951211

[B30] Secker C. Brosnan S. M. Luxenhofer R. Schlaad H. (2015). Poly(α-Peptoid)s revisited: synthesis, properties, and use as biomaterial. Macromol. Biosci. 15, 881–891. 10.1002/mabi.201500023 25851782

[B31] Shelton P. T. Jensen K. J. (2013). Linkers, resins, and general procedures for solid-phase peptide synthesis. Methods Mol. Biol. 1047, 23–41. 10.1007/978-1-62703-544-6_2 23943476

[B32] Sherrington D. C. (1990). Solid Phase Peptide Synthesis—A Practical Approach. Oxford University Press (OUP).

[B33] Shin S. B. Y. Yoo B. Todaro L. J. Kirshenbaum K. (2007). Cyclic peptoids. J. Am. Chem. Soc. 129, 3218–3225. 10.1021/ja066960o 17323948

[B34] Steinstraesser L. Hirsch T. Schulte M. Kueckelhaus M. Jacobsen F. Mersch E. A.. (2012). Innate defense regulator peptide 1018 in wound healing and wound infection. PLoS ONE 7:e39373. 10.1371/journal.pone.0039373 22879874 PMC3412849

[B35] Strøm M. B. Svendsen J. S. Rekdal O. (2000). Antibacterial activity of 15-residue lactoferricin derivatives. J. Pept. Res. 56, 265–274. 10.1034/j.1399-3011.2000.00770.x 11095180

[B36] Tran H. Gael S. L. Connolly M. D. Zuckermann R. N. (2011). Solid-phase submonomer synthesis of peptoid polymers and their self-assembly into highly-ordered nanosheets. J. Vis. Exp. e3373. 10.3791/3373 22083233 PMC3308608

[B37] Turkett J. A. Bicker K. L. (2017). Evaluating the effect of peptoid lipophilicity on antimicrobial potency, cytotoxicity, and combinatorial library design. ACS Comb. Sci. 19, 229–233. 10.1021/acscombsci.7b00007 28291947 PMC5485666

[B38] Uno T. Beausoleil E. Goldsmith R. A. Levine B. H. Zuckermann R. N. (1999). New submonomers for poly N-substituted glycines (peptoids). Tetrahedron Lett. 40, 1475–1478. 10.1016/S0040-4039(98)02696-3

[B39] Vang Mouritzen M. Jenssen H. (2018). Optimized scratch assay for *in vitro* testing of cell migration with an automated optical camera. J. Vis. Exp. 57691. 10.3791/57691 30148500 PMC6126681

[B40] Vogel H. J. Schibli D. J. Jing W. Lohmeier-Vogel E. M. Epand R. F. Epand R. M. (2002). Towards a structure-function analysis of bovine lactoferricin and related tryptophan- and arginine-containing peptides. Biochem. Cell Biol. 80, 49–63. 10.1139/o01-213 11908643

[B41] Walker J. M. (2012). Methods in molecular biology™. Methods Mol. Biol. Protoc. 925, 23–539. 10.3109/9781420019957-3

[B42] Wang Z. Shen Y. Haapasalo M. (2017). Antibiofilm peptides against oral biofilms. J. Oral Microbiol. 9:1327308. 10.1080/20002297.2017.1327308 28748031 PMC5508375

[B43] Yau W.-M. Wimley W. C. Gawrisch K. White S. H. (1998). The preference of tryptophan for membrane interfaces. Biochemistry 37, 14713–14718. 10.1021/bi980809c 9778346

[B44] Zuckermann R. N. Kerr J. M. Moosf W. H. Kent S. B. H. (1992). Efficient method for the preparation of peptoids [Oligo(N-substituted glycines)] by submonomer solid-phase synthesis. J. Am. Chem. Soc. 114, 10646–10647. 10.1021/ja00052a076

