# Supplementary material for: Synthesis of Peptoids Containing Multiple Nhtrp and Ntrp Residues: A Comparative Study of Resin, Cleavage Conditions and Submonomer Protection
Source: Front Chem. 2020 Apr 29;8:370. doi: 10.3389/fchem.2020.00370 (PMC7201050; doi:10.3389/fchem.2020.00370)
Supplement: Supplementary file 1 [file Table_1.docx]

Supplementary Material

Synthesis of peptoids containing multiple *N*htrp and *N*trp residues: A comparative study of resin, cleavage conditions and submonomer protection

Abdullah Lone^1,2^, Anis Arnous^1^, Paul Robert Hansen^2^, Biljana Mojsoska^1*^, Håvard Jenssen^1^

^1^Department of Science and Environment, Roskilde University, 4000 Roskilde, Denmark; [alone@ruc.dk](mailto:alone@ruc.dk) (AL); [an.arnous@gmail.com](mailto:an.arnous@gmail.com) (AA); [biljana@ruc.dk](mailto:biljana@ruc.dk) (BM); [jenssen@ruc.dk](mailto:jenssen@ruc.dk) (HJ)

^2^Department of Drug Design and Pharmacology, Faculty of Health and Medical Sciences, University of Copenhagen, Universitetsparken 2, 2100 Copenhagen, Denmark; [prh@sund.ku.dk](mailto:prh@sund.ku.dk) (PRH)

***Correspondence:**Biljana Mojsoska
biljana@ruc.dk

# Content

**Supplementary Tables**

**Page 4: Table S1.** RP-HPLC-ESI-MS analysis 2-9 mer sequences of Peptoid 1 synthesized using manual protocol.

**Page 5-6: Table S2.** Total crude purity and observed base peak for all traces for Peptoids 1-4 synthesized using solid-phase automated protocol.

**Page 7: Table S3.** Observed base peak for all traces for MBHA and TentaGel S RAM resins

**Supplementary Figures**

**Page 8-9: Figure S1.** RP-HPLC-ESI-MS chromatograms of Peptoid 1 (2-9 mers) synthesized using A) MBHA resin, B) TentaGel S RAM resin by manual synthesis protocol.

**Page 10: Figure S2.** RP-HPLC-ESI-MS chromatogram of Peptoid 1 (MBHA resin, cleavage condition A, solid-phase automated synthesis protocol)

**Page 11: Figure S3.** RP-HPLC-ESI-MS chromatogram of Peptoid 1 (MBHA resin, cleavage condition B, solid-phase automated synthesis protocol)

**Page 12: Figure S4.** RP-HPLC-ESI-MS chromatogram of Peptoid 2 (MBHA resin, cleavage condition A, solid-phase automated synthesis protocol)

**Page 13: Figure S5.** RP-HPLC-ESI-MS chromatogram of Peptoid 2 (MBHA resin, cleavage condition B, solid-phase automated synthesis protocol)

**Page 14: Figure S6.** RP-HPLC-ESI-MS chromatogram of Peptoid 3 (MBHA resin, cleavage condition A, solid-phase automated synthesis protocol)

**Page 15: Figure S7.** RP-HPLC-ESI-MS chromatogram of Peptoid 3 (MBHA resin, cleavage condition B, solid-phase automated synthesis protocol)

**Page 16: Figure S8.** RP-HPLC-ESI-MS chromatogram of Peptoid 4 (MBHA resin, cleavage condition A, solid-phase automated synthesis protocol)

**Page 17: Figure S9.** RP-HPLC-ESI-MS chromatogram of Peptoid 4 (MBHA resin, cleavage condition B, solid-phase automated synthesis protocol)

**Page 18: Figure S10.** RP-HPLC-ESI-MS chromatogram of Peptoid 1 (TentaGel S RAM resin, cleavage condition A, solid-phase automated synthesis protocol)

**Page 19: Figure S11.** RP-HPLC-ESI-MS chromatogram of Peptoid 1 (TentaGel S RAM resin, cleavage condition B, solid-phase automated synthesis protocol)

**Page 20: Figure S12.** RP-HPLC-ESI-MS chromatogram of Peptoid 2 (TentaGel S RAM resin, cleavage condition A, solid-phase automated synthesis protocol)

**Page 21: Figure S13.** RP-HPLC-ESI-MS chromatogram of Peptoid 2 (TentaGel S RAM resin, cleavage condition B, solid-phase automated synthesis protocol)

**Page 22: Figure S14.** RP-HPLC-ESI-MS chromatogram of Peptoid 3 (TentaGel S RAM resin, cleavage condition A, solid-phase automated synthesis protocol)

**Page 23: Figure S15.** RP-HPLC-ESI-MS chromatogram of Peptoid 3 (TentaGel S RAM resin, cleavage condition B, solid-phase automated synthesis protocol)

**Page 24: Figure S16.** RP-HPLC-ESI-MS chromatogram of Peptoid 4 (TentaGel S RAM resin, cleavage condition A, solid-phase automated synthesis protocol)

**Page 25: Figure S17.** RP-HPLC-ESI-MS chromatogram of Peptoid 4 (TentaGel S RAM resin, cleavage condition B, solid-phase automated synthesis protocol)

# Supplementary Tables

Table S1. RP-HPLC-ESI-MS analysis of observed impurities (traces) in 2-9 mer sequences of Peptoid 1 (Figure 1A) using Rink amide MBHA and TentaGel S RAM resins^3^.

| **Peptoid length mer** | **Area of traces (%)^1^** | **Corresponding *m/z* base peak signal^2^** | **Peptoid length mer** | **Area of traces (%)^1^** | **Corresponding *m/z* base peak signal^2^** |
| --- | --- | --- | --- | --- | --- |
|  |  |  |  |  |  |
|  | **MBHA** |  |  | **TentaGel S RAM** |  |
|  | | |  | | |
| **2 mer** | 62.9 | 591.4 | **2 mer** | 27.6 | 328.2 |
|  |  |  |  | 16.3 | 591.4 |
| **3 mer** | 43.3 | 591.4 | **3 mer** | 30.3 | 609.4 |
|  | 29.3 | 919.7 |  | 17.2 | 328.2 |
|  |  |  |  | 9.1 | 591.3 |
| **4 mer** | 33.7 | 591.4 | **4 mer** | 14.2 | 328.2 |
|  | 23.1 | 897.6 |  | 10.8 | 733.5 |
|  |  |  |  | 6.1 | 591.4 |
| **5 mer** | 18.6 | 591.4 | **5 mer** | 11.6 | 933.6 |
|  | 15.0 | 897.6 |  |  |  |
| **6 mer** | 19.2 | 897.6 | **6 mer** | 6.0 | 328.2 |
|  | 12.4 | 591.4 |  |  |  |
| **7 mer** | 22.5 | 591.4 | **7 mer** | 8.7 | 1173.8 |
|  | 21.5 | 919.7 |  | 6.3 | 591.4 |
| **8 mer** | 25.4 | 897.6 | **8 mer** | - | - |
|  | 24.8 | 591.4 |  |  |  |
|  | 4.9 | 978.2 |  |  |  |
| **9 mer** | 31.0 | 591.4 | **9 mer** | 8.8 | 328.2 |
|  | 31.0 | 919.7 |  | 7.1 | 919.7 |
|  |  |  |  | 6.6 | 591.4 |

^1^ Analytical RP-HPLC-ESI-MS conditions: C18 Kinetex 100 × 2.1 mm 100 Å column, 40 °C, linear gradient of 5-65 % water in acetonitrile (0.1% HCOOH) , flow rate 0.5 mL/min. Crude purity calculated with peak detection integration method ICIS (FreeStyle 1.5, Thermo Scientific) where peaks with areas more than 5% were integrated. Percentile crude purity of each length mer is shown independently of the previous length mer purity.

^2^ Observed masses of [M+2H+]2^+^, M+H^+^ or Na^+^, K^+^ adduct

^3^ Data from Figure S1 was used to construct this table

**Table S2.** Total crude purity (%) and observed base peak for all traces for Peptoids 1-4 synthesized on automated solid-phase peptide synthesizer using two different resins and cleavage conditions A and B is shown^5^.

| **Peptoid 1** | **Area of traces (%)^1^** | | | | **Corresponding *m/z* base peak signal^2^** | | | |
| --- | --- | --- | --- | --- | --- | --- | --- | --- |
|  | **MBHA** | | **TentaGel S RAM** | | **MBHA** | | **TentaGel S RAM** | |
| Trace # | A**^3^** | B**^4^** | A**^3^** | B**^4^** | A**^3^** | B**^4^** | A**^3^** | B**^4^** |
| 1 | 31 | 49 | 30 | 22 | 1368.42 (1330.42,1352.42) | 1368.5 (1330.42,1352.42) | 328.08 | 1155.33 |
| 2 | 26 | 28 | 18 | 16 | 986.25  (1008.25, 1024.25) | 625.25 | 1155.33 | 591.25 |
| 3 | 14 | 12 | 15 | 8 | 625.25  (603.25,641.17) | 1008.33  (986.25, 1024.17) | 591.25 | 919.33 |
| 4 | 7 | 5 | 8 |  | 591.25 | 591.17 | 919.25 |  |
| 5 | 6 |  |  |  | 1352.5  (1368.42) |  |  |  |
| 6 | 6 |  |  |  | 919.33 |  |  |  |
| 7 | 5 |  |  |  | 605.25 |  |  |  |
| **Peptoid 2** |  |  |  |  |  |  |  |  |
| Trace # |  |  |  |  |  |  |  |  |
| 1 | 42 | 41 | 21 | 16 | 591.25 | 591.25 | 1133.33 | 1155.33 |
| 2 | 20 | 19 | 19 | 14 | 1133.33  (1155.33,1171.33) | 919.33 | 328.17 | 591.17 |
| 3 | 18 | 14 | 18 | 10 | 897.25  (919.33,935.33) | 1155.33 | 591.17 | 919.33  (935.33,1247,1263) |
| 4 |  |  | 9 |  |  |  | 919.33 |  |
| **Peptoid 3** |  |  |  |  |  |  |  |  |
| Trace # |  |  |  |  |  |  |  |  |
| 1 | 31 | 63 | 66 | 82 | 905.42  (927.42, 943.42) | 927.42 | 905.42 | 905.42 (927.42,943.42) |
| 2 | 16 | 11 | 22 | 14 | 448.17 | 583.17 | 328.08 | 583.25  (599.17) |
| 3 | 13 | 6 | 12 | 4 | 501.25 | 633.25 | 583.25 | 257  (271.08) |
| 4 | 11 |  |  |  | 583.17 |  |  |  |
| 5 | 10 |  |  |  | 431.17 |  |  |  |
| 6 | 7 |  |  |  | 927.42 |  |  |  |
| 7 | 7 |  |  |  | 796.25 |  |  |  |
| 8 | 6 |  |  |  | 633.25 |  |  |  |
| **Peptoid 4** |  |  |  |  |  |  |  |  |
| Trace # |  |  |  |  |  |  |  |  |
| 1 | 67 | 25 | 16 | 17 | 431.17 | 448.17 | 328 | 1330.33 |
| 2 | 9 | 22 | 15 | 12 | 1314.75 | 1330.42 | 970  (561.17) | 448 |
| 3 | 7 | 10 | 15 | 9 | 891.42 | 891.25 | 1314.33 (1330.42) | 891.17  (903.42) |
| 4 | 6 |  | 15 |  | 1076.5 |  | 448.17 |  |
| 5 | 4 |  | 8 |  | 561.25  (970.5) |  | 891.08 |  |

^1^ Analytical RP-HPLC-ESI-MS conditions: C18 Kinetex 100 × 2.1 mm 100 Å column, 40 °C, linear gradient of 5-65 % water in acetonitrile (0.1% HCOOH), flow rate 0.5 mL/min. Crude purity calculated with peak detection integration method ICIS (FreeStyle 1.5, Thermo Scientific) where peaks with areas more than 5% were integrated.

^2^ Observed masses of [M+2H+]2^+^, M+ H^+^ or Na^+^, K^+^ adduct

^3^ TFA:TIPS:H_2_O (v/v %, 95:2.5:2.5), 30 min, RT

^4^ TFA:DCM:anisole (v/v %, 49:49:2), 30 min, RT

^5^ Data from Figure S2-S17 was used to construct this table

Table S3. Observed base peak for all traces for Rink amide MBHA and TentaGel S RAM resins using two different cleavage conditions A and B.

|  | **MBHA  (30 min)** | | **TentaGel S RAM  (30 min)** | | **MBHA  (120 min)** | | **TentaGel S RAM  (120 min)** | |
| --- | --- | --- | --- | --- | --- | --- | --- | --- |
| **Trace #** | **A^1^** | **B^2,^*** | **A^1^** | **B^2^** | **A^1^** | **B^2^** | **A^1^** | **B^2^** |
| **1** | 927.33 |  | 328.17 | 404.17 | 509.25 | 585.33 | 328.08 | 404.17 |
| **2** | 472.25 |  | 279.08 | 328.08 | 437.17 | 513.25 | 279.00 | 271.17 |
| **3** | 437.17 |  |  | 257.08 | 257.00 | 543.17 |  | 257.08 |
| **4** | 509.25 |  |  |  |  | 446.08 |  |  |
| **5** | 257.00 |  |  |  |  | 333.08 |  |  |

* Base peaks were not observed for the MBHA sample with cleavage condition B for 30 min.

^1^ TFA:TIPS:H_2_O (v/v %, 95:2.5:2.5), 30 min, RT

^2^ TFA:DCM:anisole (v/v %, 49:49:2), 30 min, RT

#
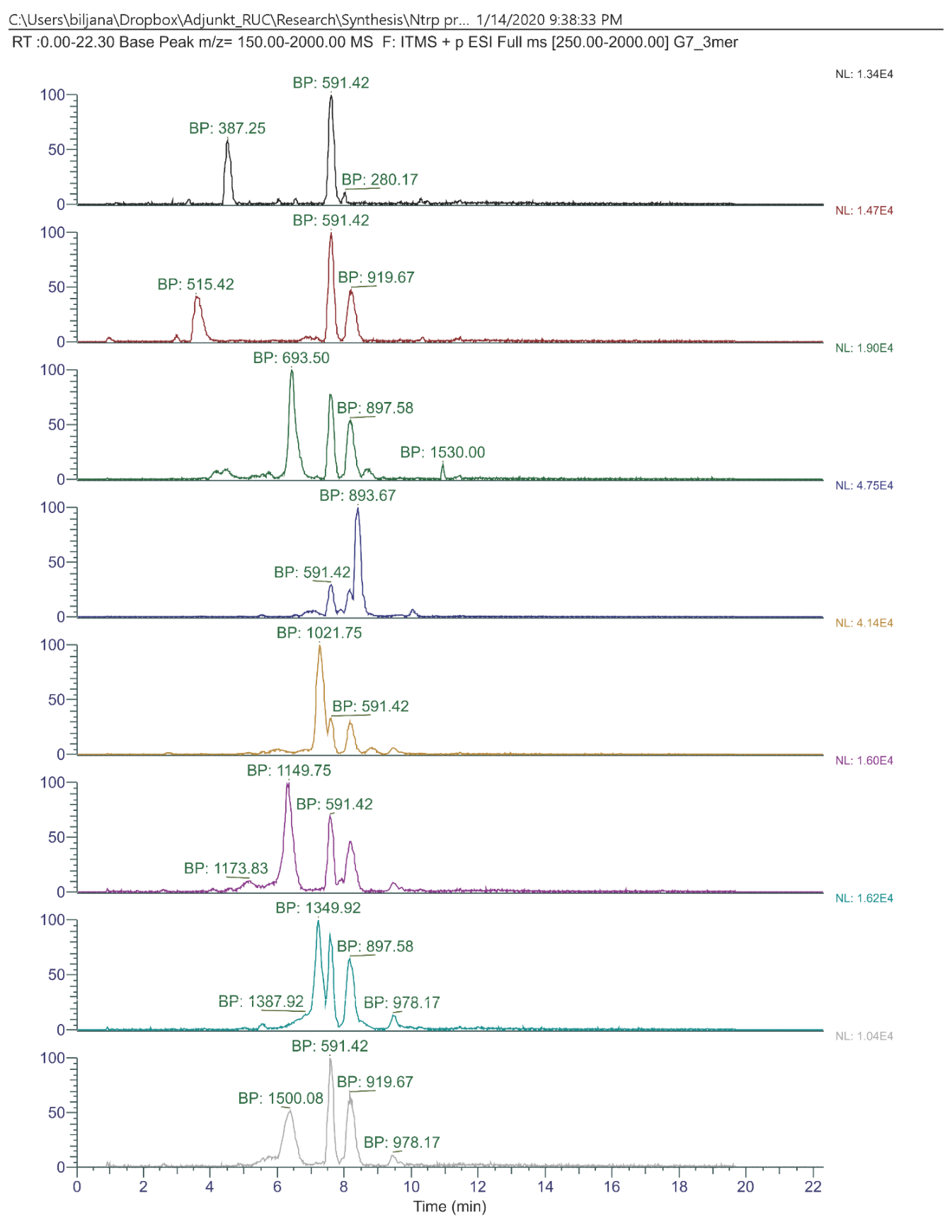
Supplementary Figures
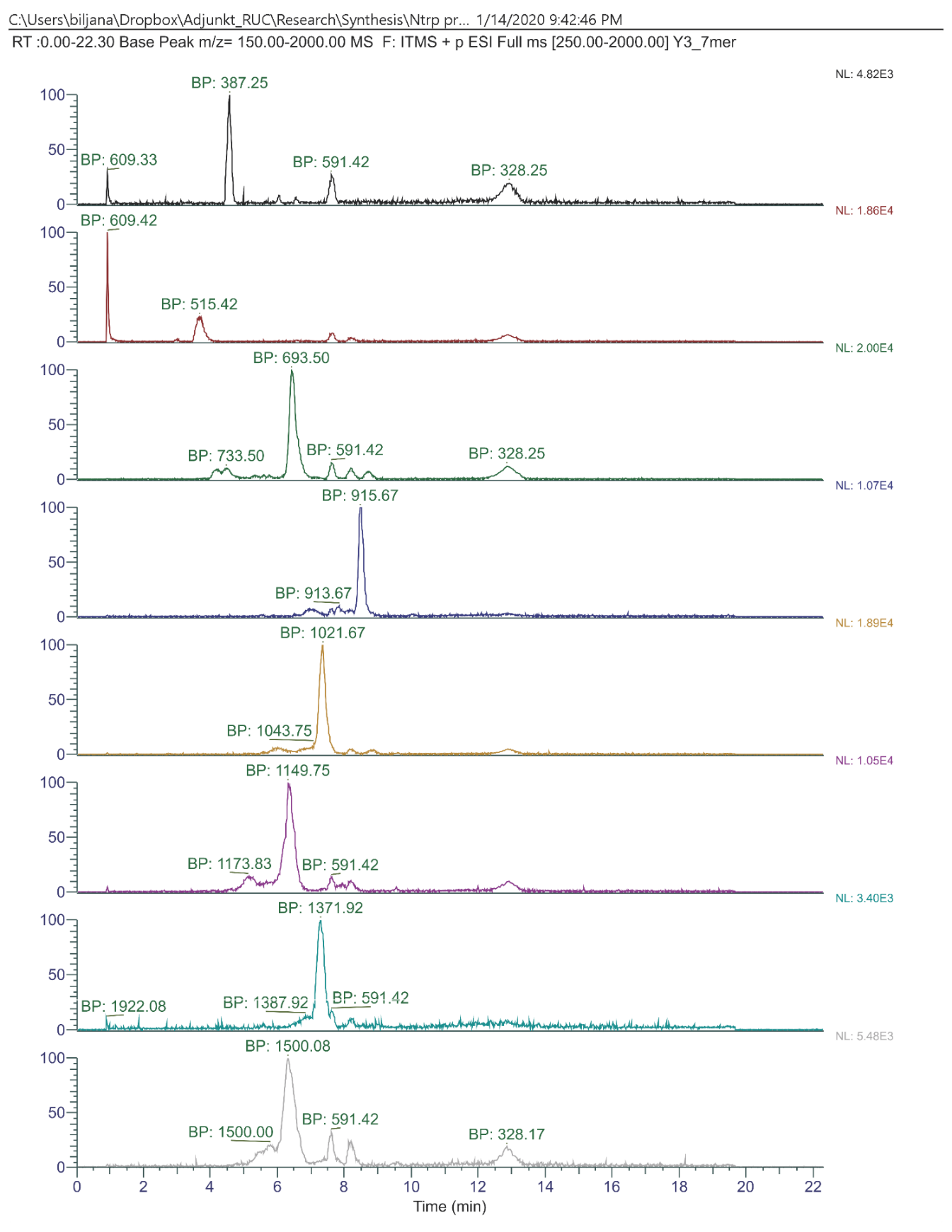


**Figure S1.** RP-HPLC-ESI-MS chromatograms of Peptoid 1 (2-9 mer) synthesized using (A) MBHA resin, (B) TentaGel S RAM resin. The chromatograms are arranged in the following order: 2 mer structure (top) 9 mer (bottom). The mass chromatograms were obtained on RP-HPLC-ESI-MS using 5-65 % acetonitrile/water gradient over 24 min using FreeStyle 1.5 software (Thermo Scientific). The most intensive m/z values observed are shown as Base Peak (BP) signals.

Figure S2. RP-HPLC-ESI-MS chromatograms of Peptoid 1 synthesized on rink amide MBHA resin and cleaved with cleavage condition A (TFA:TIPS:H_2_O, v/v % 95:2.5:2.5). The mass chromatograms were obtained using 5-65 % acetonitrile/water gradient over 24 min. The most intensive m/z values observed are shown as Base Peak (BP) signals. Chromatograms generated using FreeStyle 1.5 software (Thermo Scientific).

Figure S3. RP-HPLC-ESI-MS chromatograms of Peptoid 1 synthesized on rink amide MBHA resin and cleaved with cleavage condition B (TFA:DCM:anisole, v/v % 49:49:2). The mass chromatograms were obtained using 5-65 % acetonitrile/water gradient over 24 min. The most intensive m/z values observed are shown as Base Peak (BP) signals. Chromatograms generated using FreeStyle 1.5 software (Thermo Scientific).

Figure S4. RP-HPLC-ESI-MS chromatograms of Peptoid 2 synthesized on rink amide MBHA resin and cleaved with cleavage condition A (TFA:TIPS:H_2_O, v/v % 95:2.5:2.5). The mass chromatograms were obtained using 5-65 % acetonitrile/water gradient over 24 min. The most intensive m/z values observed are shown as Base Peak (BP) signals. Chromatograms generated using FreeStyle 1.5 software (Thermo Scientific).

Figure S5. RP-HPLC-ESI-MS chromatograms of Peptoid 2 synthesized on rink amide MBHA resin and cleaved with cleavage condition B (TFA:DCM:anisole, v/v % 49:49:2). The mass chromatograms were obtained using 5-65 % acetonitrile/water gradient over 24 min. The most intensive m/z values observed are shown as Base Peak (BP) signals. Chromatograms generated using FreeStyle 1.5 software (Thermo Scientific).

Figure S6. RP-HPLC-ESI-MS chromatograms of Peptoid 3 synthesized on rink amide MBHA resin and cleaved with cleavage condition A (TFA:TIPS:H_2_O, v/v % 95:2.5:2.5). The mass chromatograms were obtained using 5-65 % acetonitrile/water gradient over 24 min. The most intensive m/z values observed are shown as Base Peak (BP) signals. Chromatograms generated using FreeStyle 1.5 software (Thermo Scientific).

Figure S7. RP-HPLC-ESI-MS chromatograms of Peptoid 3 synthesized on rink amide MBHA resin and cleaved with cleavage condition B (TFA:DCM:anisole, v/v % 49:49:2). The mass chromatograms were obtained using 5-65 % acetonitrile/water gradient over 24 min. The most intensive m/z values observed are shown as Base Peak (BP) signals. Chromatograms generated using FreeStyle 1.5 software (Thermo Scientific).

Figure S8. RP-HPLC-ESI-MS chromatograms of Peptoid 4 synthesized on rink amide MBHA resin and cleaved with cleavage condition A (TFA:TIPS:H_2_O, v/v % 95:2.5:2.5). The mass chromatograms were obtained using 5-65 % acetonitrile/water gradient over 24 min. The most intensive m/z values observed are shown as Base Peak (BP) signals. Chromatograms generated using FreeStyle 1.5 software (Thermo Scientific).

Figure S9. RP-HPLC-ESI-MS chromatograms of Peptoid 4 synthesized on rink amide MBHA resin and cleaved with cleavage condition B (TFA:DCM:anisole, v/v % 49:49:2). The mass chromatograms were obtained using 5-65 % acetonitrile/water gradient over 24 min. The most intensive m/z values observed are shown as Base Peak (BP) signals. Chromatograms generated using FreeStyle 1.5 software (Thermo Scientific).

Figure S10. RP-HPLC-ESI-MS chromatograms of Peptoid 1 synthesized on rink amide TentaGel S RAM resin and cleaved with cleavage condition A (TFA:TIPS:H_2_O, v/v % 95:2.5:2.5). The mass chromatograms were obtained using 5-65 % acetonitrile/water gradient over 24 min. The most intensive m/z values observed are shown as Base Peak (BP) signals. Chromatograms generated using FreeStyle 1.5 software (Thermo Scientific).

Figure S11. RP-HPLC-ESI-MS chromatograms of Peptoid 1 synthesized on rink amide TentaGel S RAM resin and cleaved with cleavage condition B (TFA:DCM:anisole, v/v % 49:49:2). The mass chromatograms were obtained using 5-65 % acetonitrile/water gradient over 24 min. The most intensive m/z values observed are shown as Base Peak (BP) signals. Chromatograms generated using FreeStyle 1.5 software (Thermo Scientific).

Figure S12. RP-HPLC-ESI-MS chromatograms of Peptoid 2 synthesized on rink amide TentaGel S RAM resin and cleaved with cleavage condition A (TFA:TIPS:H_2_O, v/v % 95:2.5:2.5). The mass chromatograms were obtained using 5-65 % acetonitrile/water gradient over 24 min. The most intensive m/z values observed are shown as Base Peak (BP) signals. Chromatograms generated using FreeStyle 1.5 software (Thermo Scientific).

Figure S13. RP-HPLC-ESI-MS chromatograms of Peptoid 2 synthesized on rink amide TentaGel S RAM resin and cleaved with cleavage condition B (TFA:DCM:anisole, v/v % 49:49:2). The mass chromatograms were obtained using 5-65 % acetonitrile/water gradient over 24 min. The most intensive m/z values observed are shown as Base Peak (BP) signals. Chromatograms generated using FreeStyle 1.5 software (Thermo Scientific).

Figure S14. RP-HPLC-ESI-MS chromatograms of Peptoid 3 synthesized on rink amide TentaGel S RAM resin and cleaved with cleavage condition A (TFA:TIPS:H_2_O, v/v % 95:2.5:2.5). The mass chromatograms were obtained using 5-65 % acetonitrile/water gradient over 24 min. The most intensive m/z values observed are shown as Base Peak (BP) signals. Chromatograms generated using FreeStyle 1.5 software (Thermo Scientific).

Figure S15. RP-HPLC-ESI-MS chromatograms of Peptoid 3 synthesized on rink amide TentaGel S RAM resin and cleaved with cleavage condition B (TFA:DCM:anisole, v/v % 49:49:2). The mass chromatograms were obtained using 5-65 % acetonitrile/water gradient over 24 min. The most intensive m/z values observed are shown as Base Peak (BP) signals. Chromatograms generated using FreeStyle 1.5 software (Thermo Scientific).

Figure S16. RP-HPLC-ESI-MS chromatograms of Peptoid 4 synthesized on rink amide TentaGel S RAM resin and cleaved with cleavage condition A (TFA:TIPS:H_2_O, v/v % 95:2.5:2.5). The mass chromatograms were obtained using 5-65 % acetonitrile/water gradient over 24 min. The most intensive m/z values observed are shown as Base Peak (BP) signals. Chromatograms generated using FreeStyle 1.5 software (Thermo Scientific).

Figure S17. RP-HPLC-ESI-MS chromatograms of Peptoid 4 synthesized on rink amide TentaGel S RAM resin and cleaved with cleavage condition B (TFA:DCM:anisole, v/v % 49:49:2). The mass chromatograms were obtained using 5-65 % acetonitrile/water gradient over 24 min. The most intensive m/z values observed are shown as Base Peak (BP) signals. Chromatograms generated using FreeStyle 1.5 software (Thermo Scientific).
